# Insecticidal Activity of *Plectranthus amboinicus* Essential Oil against the Stable Fly *Stomoxys calcitrans* (Diptera: Muscidae) and the Horse Fly *Tabanus megalops* (Diptera: Tabanidae)

**DOI:** 10.3390/insects13030255

**Published:** 2022-03-03

**Authors:** Arpron Leesombun, Sivapong Sungpradit, Sookruetai Boonmasawai, Thekhawet Weluwanarak, Suriyo Klinsrithong, Jiraporn Ruangsittichai, Sumate Ampawong, Roungthip Masmeatathip, Tanasak Changbunjong

**Affiliations:** 1 Department of Pre-Clinic and Applied Animal Science, Faculty of Veterinary Science, Mahidol University, Nakhon Pathom 73170, Thailand; arpron.lee@mahidol.edu (A.L.); sivapong.sun@mahidol.edu (S.S.); sookruetai.boo@mahidol.edu (S.B.); 2 The Monitoring and Surveillance Center for Zoonotic Diseases in Wildlife and Exotic Animals (MoZWE), Faculty of Veterinary Science, Mahidol University, Nakhon Pathom 73170, Thailand; thekhawet.wel@mahidol.edu; 3 The Center of Veterinary Diagnosis, Faculty of Veterinary Science, Mahidol University, Nakhon Pathom 73170, Thailand; suriyo.kli@mahidol.ac.th; 4 Department of Medical Entomology, Faculty of Tropical Medicine, Mahidol University, Bangkok 10400, Thailand; jiraporn.rua@mahidol.ac.th; 5 Department of Tropical Pathology, Faculty of Tropical Medicine, Mahidol University, Bangkok 10400, Thailand; am_sumate@hotmail.com; 6 Department of Entomology, Faculty of Agriculture at Kamphaeng Saen, Kamphaeng Saen Campus, Kasetsart University, Nakhon Pathom 73140, Thailand; fagrrtm@ku.ac.th

**Keywords:** contact toxicity, fumigant toxicity, horse flies, insecticide, *Plectranthus amboinicus*, stable flies, vector

## Abstract

**Simple Summary:**

*Plectranthus amboinicus* (Lour.) Spreng., commonly known as Indian borage, has been reported to have insecticidal activity against various insects. In this study, the insecticidal properties (contact and fumigant toxicities) derived from *P. amboinicus* essential oil were investigated against the stable fly, *Stomoxys calcitrans*, and the horse fly, *Tabanus megalops*. The results showed that *P. amboinicus* essential oil has both contact and fumigant toxicities against the target species and thus has potential as an alternative control agent.

**Abstract:**

The stable fly, *Stomoxys calcitrans* (Diptera: Muscidae), and the horse fly, *Tabanus megalops* (Diptera: Tabanidae), are important ectoparasites of livestock in Thailand. These species affect animal health and cause economic losses. This study investigated the insecticidal activity of *Plectranthus amboinicus* essential oil against *S. calcitrans* and *T. megalops* through contact and fumigant toxicity tests and evaluated the effects of the essential oil on these flies through histopathological and scanning electron microscopic (SEM) studies. The results of the contact toxicity test indicated that the median lethal dose against *S. calcitrans* and *T. megalops* was 12.05 and 131.41 µg/fly, and the 90% lethal dose was 45.53 and 200.62 µg/fly, respectively. The results of the fumigant toxicity test showed that the median lethal concentration against *S. calcitrans* and *T. megalops* was 1.34 and 7.12 mg/L air, and the 90% lethal concentration was 4.39 and 30.37 mg/L air, respectively. Histopathology revealed neuronal degeneration in the brain of *S. calcitrans* and interstitial neuronal edema of the brain and ovarian necrosis in *T. megalops.* No external morphological changes were observed via SEM. Given its insecticidal properties against *S. calcitrans* and *T. megalops*, *P. amboinicus* essential oil could be developed into a natural insecticide to control these fly species.

## 1. Introduction

Stable flies (*Stomoxys* spp. (Diptera: Muscidae)) and horse flies (*Tabanus* spp. (Diptera: Tabanidae)) are blood-sucking parasites of animals and humans. They are considered as important pests of livestock in Thailand, causing direct problems in animal health especially with horses, cattle, and buffaloes, and considerable economic losses [1,2,3,4]. Annual economic losses due to stable flies are estimated to be more than USD 2 billion in the United States [5]. Both flies can cause skin irritation, blood loss, decreased grazing efficiency, reduced weight gain, and decreased milk production [6,7]. They also serve as vectors of several animal pathogens, including bacteria, helminths, protozoa, and viruses [6,7]. In Thailand, these fly species are important mechanical vectors of *Trypanosoma evansi* (Trypanosomatida: Trypanosomatidae), the protozoan pathogen causing trypanosomosis or surra in horses, cattle, and buffaloes, resulting in an acute, subacute, or chronic disease in these animals [3]. Moreover, both the stable fly and the horse fly can mechanically transmit numerous pathogens in cattle such as *Anaplasma marginale* (Rickettsiales: Anaplasmataceae), bovine leukemia virus, and lumpy skin disease virus [6,7,8].

Currently, several chemical products are used to control stable flies [9,10]. However, the repeated use of chemicals causes insects to develop resistance to insecticides, leading to adverse effects on human and environmental health. *Stomoxys calcitrans* is reportedly resistant to organophosphates and pyrethroids [11,12,13]. The use of natural products, especially plant essential oils, as an alternative to chemical agents has attracted increasing interest because they are safe and exhibit biological activity against insects [14,15].

Thailand is rich in natural resources, and many herbal plants have been used in traditional medicine for treating common ailments [16,17]. The essential oils of some herbal plants contain various secondary metabolites that protect against natural pests. The most prevalent among these secondary metabolites are alkaloids, saponins, phenols, and terpenes, all of which possess insecticidal properties [15]. Many plant families produce essential oils that can control insect pests. For instance, Asteraceae is effective against the larval and adult stages of *Aedes aegypti* mosquitoes [18], whereas Rutaceae is effective against the larval and adult stages of blow flies (*Chrysomya megacephala*, *C. rufifacies,* and *Lucilia cuprina*) and the house fly (*Musca domestica*) [19]. Lamiaceae is an important family producing essential oils with insecticidal activities [20].

*Plectranthus amboinicus* (Lour.) Spreng. (synonyms: *Coleus amboinicus* Lour. and *C. aromaticus* Benth.), commonly known as Indian borage (Thai name: Niam hu suea), is a perennial herb belonging to the family Lamiaceae and is widely distributed throughout the tropics and warm regions of the Old World, in particular Africa, Asia, and Australia [21]. This herb exerts numerous pharmacological properties, including antimicrobial, anti-inflammatory, antitumor, wound-healing, anti-epileptic, insecticidal, antioxidant, and analgesic activities [21,22,23]. The essential oil of this herb contains two major phenolic monoterpenes, namely carvacrol and thymol, which show insecticidal activity [24,25]. The essential oil from *P. amboinicus* exhibits insecticidal activity against various insects, including cowpea weevil (*Callosobruchus maculatus*) [25], mosquito larvae (*Ae. aegypti* and *Anopheles gambiae*) [26,27], red flour beetle (*Tribolium castaneum*) [28], and the termite (*Odontotermes obesus*), among others [29]. However, the efficacy of *P. amboinicus* essential oil against stable flies and horse flies has not yet been reported. Accordingly, the present study assessed the insecticidal activity of the essential oil from *P. amboinicus* against *S. calcitrans* and *T. megalops* by contact and fumigant toxicity tests and to evaluate the effects of the essential oil on these flies through histopathological test and scanning electron microscopy (SEM).

## 2. Materials and Methods

### 2.1. Ethical Statement

The study protocol was approved by the Faculty of Veterinary Science, Mahidol University Animal Care and Use Committee (Ref. MUVS-2020-12-63).

### 2.2. Insects

Populations of *S. calcitrans* and *T. megalops* were collected from a horse farm in Nakhon Pathom Province, central Thailand (13°45′43.4′′ N 100°08′15.7′′ E), between March and May 2021, by using Nzi Traps [30,31]. Farmers in this farm reported that they never used insecticides. The traps were placed at the collection site from 16:00 to 19:00. The flies were stored in plastic cups and then transported within Styrofoam boxes containing ice packs to the Pharmacology Laboratory, Faculty of Veterinary Science, Mahidol University. After being transported to the laboratory, the flies were maintained at 27–29 °C and 70–80% relative humidity until testing time. Males and females of *S. calcitrans* and females of *T. megalops* were used for testing. They were selected from groups of undamaged and unfed flies [32] under a stereomicroscope (SMZ745, Nikon, Tokyo, Japan) without anesthesia.

### 2.3. Essential Oil Extraction and Quantification

**Figure 1:**
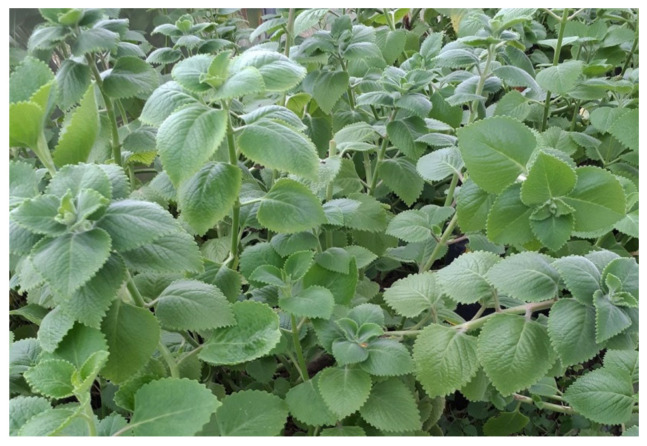
*Plectranthus amboinicus* (Lour.) Spreng. used in this study.

*P. amboinicus* (Figure 1) was obtained from a pesticide-free garden in Nonthaburi Province, central Thailand (13°51′43.8′′ N 100°24′33.8′′ E). The plant was identified and deposited at the Department of Pharmaceutical Botany, Faculty of Pharmacy, Mahidol University (PBM No.005507-8). Essential oil was extracted from 10 kg of fresh leaves through steam distillation for 6 h. The essential oil obtained was stored in amber glass bottles at 4 °C until used. Essential oil yield was expressed in % (*v*/*w*), based on the weight of the fresh plant material.

The physical properties of the essential oil were analyzed as follows. The color was determined through visual inspection; pH was measured with pH-indicator strips (Merck, Darmstadt, Germany); density was measured with a density meter (DA-100M, Tokyo, Japan); and the refractive index was calculated using a refractometer (RX-5000CX, Atago, Tokyo, Japan). The physical properties are significant to evaluate the quality of the essential oil and can be used as criteria for its identification.

The chemical components of the essential oil were analyzed using gas chromatography–mass spectrometry (GC-MS) (model 7890A-MS5975C, Agilent Technologies, Santa Clara, CA, USA) equipped with a DB-5HT capillary column (length: 30 m, inner diameter 0.25 mm, and film thickness 0.1 µm, Agilent Technologies, USA). The sample was injected in split mode with a 1:10 split ratio. Helium was used as the carrier gas at a flow rate of 1 mL min^−1^. The temperature of the injection port was 250 °C, and the column temperature program was as follows: 50 °C for 2 min, followed by an increase to 250 °C at a rate of 10 °C min^−1^, after which the temperature was maintained at 250 °C for 5 min. The MS conditions included an ion source temperature of 230 °C, an ionization energy of 70 eV, and a mass scan range of 350–550 amu. The components were identified by comparing their mass spectra with data in Wiley 7N edition (Mass Spectra library). The concentration of major components was calculated by comparing the peak area of the sample with the peak area of the standard.

### 2.4. Contact Toxicity Test

Contact toxicity test of the essential oil from *P. amboinicus* against *S. calcitrans* and *T. megalops* was performed by topical application in accordance with the procedure described by Zhu et al., (2011) [33] with certain modifications. Preliminary studies were carried out to determine the appropriate concentration ranges (10–90% mortality). Essential oil concentrations of *P. amboinicus* were immediately prepared in 1.5 mL microcentrifuge tubes using acetone as the solvent. Five concentrations of essential oil were used in this test: *S. calcitrans* (mixed sexes) with 9.3, 18.7, 37.4, 46.7, and 93.4 µg/µL and *T. megalops* (females only) with 93.4, 116.8, 140.1, 163.5, and 186.8 µg/µL. The flies were anesthetized at −20 °C for 30–45 s, and 0.5–1 µL (0.5 µL for *S. calcitrans*; 1 µL for *T. megalops*) of each concentration was applied directly on the thorax using a micropipette. Acetone and cypermethrin (1% *w*/*v*) were applied in the same volume as negative and positive controls, respectively. Each treatment was carried out with 10 flies in three replications. After topical application, the treated flies were placed in a sterile clear plastic cup of 11 cm diameter and 8.5 cm height (*S. calcitrans*: 10 flies/cup and *T. megalops*: 5 flies/cup), and the cup was covered with a mesh fabric and secured with rubber bands. Honey solution (10%) on cotton wool was provided as an energy source of the flies at the top of the mesh fabric. The flies were allowed to recover at 27–29 °C and 70–80% relative humidity. Mortality was recorded at 1, 2, 4, 6, 12, and 24 h after treatment. The flies were considered dead when they did not move after mechanical stimulation with a paintbrush.

### 2.5. Fumigant Toxicity Test

Fumigant toxicity test of the essential oil from *P. amboinicus* against *S. calcitrans* and *T. megalops* was conducted as previously described by Zhu et al., (2011) [33], with certain modifications. This test was performed in a 1 L sterile clear plastic box with a lid. Preliminary studies were carried out to determine the appropriate concentration ranges (10–90% mortality). Different amounts of essential oil (0.47, 0.93, 1.87, 2.80, and 4.67 mg for *S. calcitrans*; 2.34, 4.67, 9.34, 18.68, and 28.02 mg for *T. megalops*) dissolved in 100 µL of acetone were separately pipetted onto 55 mm diameter Whatman no. 1 filter papers (GE Healthcare, Buckinghamshire, UK), and placed onto the bottom of a glass Petri dish (diameter 55 mm). The solvent on each piece of filter paper (in the Petri dish) was allowed to evaporate for 2–3 min, after which the Petri dish was covered with mesh fabric and secured with rubber bands to prevent contact between the filter paper and the flies. Acetone and cypermethrin (1% *w*/*v*) were used as negative and positive controls, respectively. The Petri dishes were placed on the bottom of a plastic box. Honey solution (10%) on cotton wool was also placed at the bottom of the plastic box. Mixed sexes of *S. calcitrans* and females of *T. megalops* were used for testing. The flies were anesthetized at −20 °C for 30–45 s and placed in the plastic box, and each box was closed. Each treatment was carried out with 10 flies in three replications. The flies were allowed to recover and maintained at 27–29 °C with 70–80% relative humidity. Mortality was recorded at 1, 2, 4, 6, 12, and 24 h after treatment. The flies were considered dead when they did not move.

### 2.6. Histopathological Study

Histopathological analysis was conducted after *S. calcitrans* and *T. megalops* were exposed to the median lethal dose (*S. calcitrans*: 12.05 µg/fly; *T. megalops*: 131.41 µg/fly) of the essential oil at 24 h. Three dead specimens (*S. calcitrans*: 2 females and 1 male; *T. megalops*: 3 females) and control specimens of each species were used in this study. The specimens were fixed in 10% neutral buffer formalin for 7 days, dehydrated through graded ethanol, and then infiltrated and embedded in paraffin. Each specimen was sliced into 4 µm sections using a rotary microtome (LEICA RM2125; Leica Microsystems, Nussloch, Germany) and then stained with hematoxylin and eosin. Histopathological changes were examined under a light microscope (ECLIPSE Ei, Nikon, Tokyo, Japan) by focusing on the musculoskeletal, gastrointestinal, urinary, reproductive, and nervous systems.

### 2.7. Scanning Electron Microscopic Study

Ultrastructural changes in the external morphology of *S. calcitrans* and *T. megalops* exposed to a median lethal dose (*S. calcitrans*: 12.05 µg/fly; *T. megalops*: 131.41 µg/fly) of the essential oil at 24 h were investigated. Three dead specimens and control specimens of each species were used in this study. The specimens were immersed in a primary fixative with 2.5% glutaraldehyde and a secondary fixative with 1% osmium tetroxide. Each specimen was dehydrated through graded ethanol, dried in a critical dryer (HCP-2; HITACHI, Tokyo, Japan), and then stubbed and coated with sputter coater (EMITECH K550, Emitech Ltd., Ashford, UK). Morphological changes were examined under a scanning electron microscope (JSM-6610LV, JEOL, Tokyo, Japan).

### 2.8. Data Analysis

Toxicity tests characterized by more than 20% of control mortality were discharged and repeated. When control mortality was greater than 5%, the observed mortality was corrected using the Abbott’s formula [34]. All data were checked for normality and homogeneity of the variance with Shapiro–Wilk and Levene tests, respectively. Statistical comparisons of the mortality between treatments were analyzed using one-way ANOVA followed by Tukey’s HSD test in SPSS version 21.0 software (SPSS, Chicago, IL, USA). The effects of the treatments and exposure times on the mortality were analyzed using repeated measures ANOVA and Greenhouse–Geisser correction in SPSS version 21.0 software. The repeated factor was exposure time, while the response variable was insect mortality and the main effect was treatment. Values of *p* < 0.05 were considered significant. Toxicity values, including median lethal dose (LD_50_) and 90% lethal dose (LD_90_) at 24 h after treatment and median lethal concentrations (LC_50_) and 90% lethal concentration (LC_90_) at 24 h after treatment, were calculated using probit analysis in LdP line software (Ehab Mostafa Bakr, Dokki, Cairo, Egypt), which can be downloaded for free at http://www.ehabsoft.com/ldpline/, accessed on 1 November 2021.

## 3. Results

### 3.1. Essential Oil Extraction and Quantification

The yield of *P. amboinicus* essential oil obtained from fresh leaves was 0.10% (*v*/*w*). The oil was clear yellow and had a pH of 6, a density of 0.93 g/mL at 20 °C, and a refractive index of 1.51. The chemical constituents of *P. amboinicus* essential oil were determined using GC-MS. A total of 17 compounds were identified, accounting for 96.71% of the total oil. The essential oil contained alpha-bergamotene (5.36%), alpha-humulene (2.04%), alpha-phellandrene (0.13%), alpha-pinene (0.11%), alpha-terpinene (1.44%), alpha-terpinolene (0.29%), beta-bisabolene (0.32%), beta-caryophyllene (6.19%), beta-farnesene (0.22%), beta-myrcene (0.69%), beta-pinene (0.04%), carvacrol (63.46%), gamma-terpinene (9.39%), p-cymene (5.27%), 1-phellandrene (0.32%), 4-terpineol (1.23%), thymol (0.21%), and unknown compounds (1.21%). The major components were carvacrol, gamma-terpinene, and beta-caryophyllene. Carvacrol was used to quantify the essential oil, and the carvacrol concentration was 0.42 % (*w*/*v*).

### 3.2. Contact Toxicity Test

The contact toxicity of the essential oil from *P. amboinicus* against *S. calcitrans* and *T. megalops* was observed among the different concentrations of essential oil at 24 h after treatment. The negative control (acetone) was used to validate the test as no insecticidal activity against the flies. In the case of *S. calcitrans*, the negative control and the essential oil at 9.3 18.7, and 37.4 µg/µL presented low or no insecticidal activity when compared with 46.7 and 93.4 µg/µL and cypermethrin (Table 1). The essential oil at 46.7 µg/µL showed insecticidal activity similar to cypermethrin at 24 h after treatment, whereas the essential oil at 93.4 µg/µL showed insecticidal activity similar to cypermethrin from 2 to 24 h after treatment. The interaction between the concentration and time was statistically significant on *S. calcitrans* mortality (time, F_(2.06, 28.79)_ = 35.08, *p* < 0.001; treatment, F_(6, 28.79)_ = 61.86, *p* < 0.001; treatment × time, F_(12.34, 28.79)_ = 3.23, *p* < 0.001). As for *T. megalops*, the negative control and all treatments presented low or no insecticidal activity when compared with cypermethrin (Table 2). The interaction between the concentration and time was statistically significant on *T. megalops* mortality (time, F_(1.78, 24.87)_ = 69.38, *p* < 0.001; treatment, F_(6, 24.87)_ = 78.67, *p* < 0.001; treatment × time, F_(10.66, 24.87)_ = 8.03, *p* < 0.001). The LD_50_ against *S. calcitrans* and *T. megalops* was 12.05 and 131.41 µg/fly, and the LD_90_ was 45.53 and 200.62 µg/fly, respectively (Table 3).

### 3.3. Fumigant Toxicity Test

The fumigant toxicity of the essential oil from *P. amboinicus* against *S. calcitrans* and *T. megalops* was observed among the different concentrations of essential oil at 24 h after treatment. The negative control (acetone) was used to validate the test as no insecticidal activity against the flies. For *S. calcitrans*, the negative control and the essential at 0.47, 0.93, and 1.87 mg/L air presented low or no insecticidal activity when compared with the essential oil at 2.80 and 4.67 mg/L air and cypermethrin (Table 4). At 2.80 and 4.67 mg/L air, the essential oil showed insecticidal activity similar to cypermethrin at 24 h after treatment. The interaction between the concentration and time was statistically significant on *S. calcitrans* mortality (time, F_(2.69, 37.60)_ = 86.02, *p* < 0.001; treatment, F_(6, 37.60)_ = 111.32, *p* < 0.001; treatment × time, F_(16.11, 37.60)_ = 12.98, *p* < 0.001). As for *T. megalops*, the negative control and the essential oil at 2.34, 4.67, 9.34, and 18.68 mg/L air resulted in low or no insecticidal activity when compared with the essential oil at 28.02 mg/L air and cypermethrin (Table 5). The essential oil at 28.02 mg/L air showed insecticidal activity similar to cypermethrin at 12 and 24 h after treatment. The interaction between the concentration and time was statistically significant on *T. megalops* mortality (time, F_(2.43, 34.02)_ = 313.34, *p* < 0.001; treatment, F_(6, 34.02)_ = 228.95, *p* < 0.001; treatment × time, F_(14.58, 34.02)_ = 41.52, *p* < 0.001). The LC_50_ against *S. calcitrans* and *T. megalops* was 1.34 and 7.12 mg/L air, and the LC_90_ was 4.39 and 30.37 mg/L air, respectively (Table 3).

### 3.4. Histopathological Study

**Figure 2:**
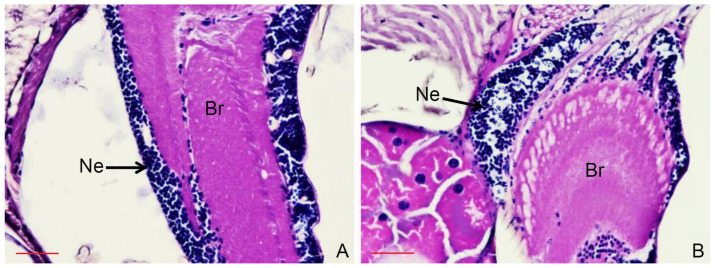
Histopathological changes in the brain of *Stomoxys calcitrans*: control (**A**) and at 24 h after treatment with *Plectranthus amboinicus* essential oil (12.05 µg/fly) showing neuronal degeneration (**B**). Light microscope image of a paraffin section stained with hematoxylin and eosin. (**A**,**B**) Bar, 50 µm. Br: brain; Ne: neurons.

**Figure 3:**
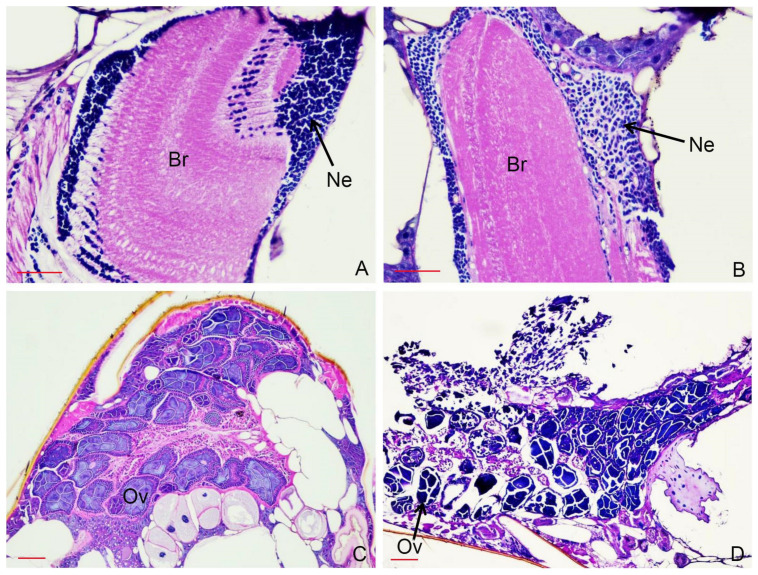
Histopathological changes in the brain (**A**,**B**) and ovaries (**C**,**D**) of *Tabanus megalops*: control (**A**,**C**) and at 24 h after treatment with *Plectranthus amboinicus* essential oil (131.41 µg/fly) showing interstitial neuronal edema of the brain (**B**) and ovarian necrosis (**D**). Light microscope image of a paraffin section stained with hematoxylin and eosin. (**A**,**B**) Bar, 50 µm. (**C**,**D**) Bar, 100 µm. Br: brain; Ne: neurons; Ov: ovaries.

A histopathological study was conducted to examine the effects of the essential oil from *P. amboinicus* on *S. calcitrans* and *T. megalops* at 24 h after treatment. Neuronal degeneration was observed in the brain of *S. calcitrans* (Figure 2), and interstitial neuronal edema of the brain and ovarian necrosis were evident in *T. megalops* (Figure 3). No histopathological changes were found in the control flies.

### 3.5. Scanning Electron Microscopy

Scanning electron microscopy was performed to examine the effects of *P. amboinicus* essential oil on the external morphology of *S. calcitrans* and *T. megalops* at 24 h after treatment. No external morphological changes were found in either the treated or control flies except the loss of some setae.

## 4. Discussion

This study demonstrated the insecticidal activity of *P. amboinicus* essential oil against the stable fly, *S. calcitrans*, and the horse fly, *T. megalops*, both of which are common species in Thailand [2,4]. One limitation of our study is the fact that the specimens were collected directly from the field rather than reared in the laboratory. Stable fly and horse fly colonies are difficult to establish and maintain in the laboratory; published reports of rearing these flies in Thailand are also lacking. Ideally, for the testing of insecticides, the specimens (F1 generation) should be obtained from laboratory colonies to limit the influence of factors such as age and physiological status [32]. However, the advantages of direct field-collected specimens are (a) convenience and (b) the results are pertinent to the target population [32]. In the present study, we selected and used only unfed flies to reduce the factor of physiological status in accordance with WHO recommendations [32]. Indeed, several studies on insecticide susceptibility used wild caught flies, such as sand flies [35,36] and stable flies [12,37].

The toxicity of an insecticide can vary depending on the mode of entry in the insect, such as through ingestion, contact, or aspiration (fumigation) [38]. In the present study, the contact and fumigant toxicity tests revealed that the *P. amboinicus* essential oil exerts lethal effects against adult *S. calcitrans* and *T. megalops* in a dose-dependent manner. The essential oil at low concentrations of 9.3, 18.7, and 37.4 µg/µL showed similar mortality to the negative control (acetone) in the contact toxicity test against *S. calcitrans*, whereas the essential oil at high doses of 46.7 and 93.4 µg/µL showed similar mortality to the positive control (cypermethrin 1%). These results are similar to the results of the contact toxicity test of tea tree (*Melaleuca alternifolia*) essential oil against *S. calcitrans*, in which little or no insecticidal effect was found at treatment concentrations of 0.5, 1, and 2.5% (*w*/*v*), and a high treatment concentration of 5% (*w*/*v*) resulted in an insecticidal effect similar to the positive control (Diazinon 1%) [39]. However, the insecticidal efficacy was rather low at the first hours of observation after the exposure and was approximately 90% at 24 h. The insecticidal efficacy of the essential oil is influenced by the dose and time of exposure. In terms of toxicity values obtained in the present study, the LD_50_ and LC_50_ of the *P. amboinicus* essential oil against *S. calcitrans* at 24 h after treatment for the contact and fumigant toxicity tests were 12.05 µg/fly or 2.41% (*w*/*v*) and 1.34 mg/L air or 1.34 µg/cm^3^ air, respectively. The efficacy of other essential oils against this species has been documented; for example, the essential oil from catnip (*Nepeta cataria*) showed LD_50_ and LC_50_ against *S. calcitrans* of 16.4 µg/fly and 10.7 mg/cm^3^, respectively [33], and the essential oil from Japanese pepper (*Zanthoxylum piperitum*) and bamboo-leaf prickly ash (*Z. armatum*) tested against *S. calcitrans* showed LD_50_ and LC_50_ of 11.058 µg/fly and 0.264 µg/cm^3^, and 26.981 µg/fly and 0.347 µg/cm^3^, respectively [40]. In addition, *M. alternifolia* essential oil tested against *S. calcitrans* in a 15 min contact and fumigant treatments showed LD_50_ and LC_50_ of 3.82 and 1.06% (*w*/*v*), respectively [39].

To the best of our knowledge, this study is the first to report the insecticidal activity of essential oil against horse flies. The contact and fumigant toxicity tests revealed that the LD_50_ and LC_50_ of *P. amboinicus* essential oil against adult *T. megalops* at 24 h after treatment were 131.41 µg/fly or 13.14% (*w*/*v*) and 7.12 mg/L air or 7.12 µg/cm^3^ air, respectively. The results indicated that the LD_50_ of *P. amboinicus* essential oil against horse flies was approximately 10 times the corresponding value in stable flies. In comparing the toxicity values of contact and fumigant treatments against *S. calcitrans*, lower toxicity levels (LD_50_, LD_90_, LC_50_, and LC_90_) were obtained from the fumigant treatment than the contact treatment. This result indicates that *P. amboinicus* essential oil is more effective as a fumigant treatment than a contact treatment. These results are consistent with the finding of Dillmann et al., (2020) [39] that *M. alternifolia* essential oil shows greater insecticidal activity when used as a fumigant rather than a topical application [39]. Meanwhile, a higher value of LC_90_ from fumigation was recorded in *T. magalops*. Therefore, a relatively high dose of *P. amboinicus* essential oil vapor is required to kill horse flies. The chemical composition of an essential oil significantly affects its insecticidal activity [41]; monoterpenes such as carvacrol and thymol are prevalent in essential oils with evident repellent and insecticide effects [42]. In the present study, the main chemical constituents of *P. amboinicus* essential oil were carvacrol (63.46%), gamma-terpinene (9.39%), and beta-caryophyllene (6.19%), whereas thymol concentration was relatively low. The difference in main chemical compositions is influenced by environmental factors, seasonality, and extraction processes [21]. Several studies have shown that carvacrol, thymol, and beta-caryophyllene are potential insecticides. Carvacrol and thymol exert insecticidal activity against *Culex pipiens* eggs and larvae [43], *Pochazia shantungensis* nymphs [44], *Cimex lectularius* [45], and *Mahanarva spectabilis* [46], whereas beta-caryophyllene (sesquiterpene group) exhibits toxicity against *Megoura japonica* and *Plutella xylostella*, with LD_50_ values of 0.072 µg/adult and 0.32 µg/larva, respectively [47]. The combination of carvacrol and thymol (ratio 4:1) exerts a synergistic effect against larvae of *Cx. pipiens*, and an additive effect has been observed on eggs [43]. Thus, the insecticidal effects of *P. amboinicus* essential oil may be due to the presence of carvacrol, beta-caryophyllene, or thymol or the combination effects of the chemical constituents. The ratio of carvacrol to thymol has been investigated as a possible synergist or additive effect [43,44,45,46].

The histopathological changes observed in the present study indicate neuronal degeneration in the brain of *S. calcitrans* and interstitial neuronal edema of the brain and ovarian necrosis in *T. megalops*. We posit that *P. amboinicus* essential oil directly affects the nervous and reproductive systems of the target insects; however, the mechanism of action should be investigated. Previous studies have shown that insecticides work by affecting different biological systems, including the nervous system, endocrine system, energy production, integument development, and water homeostasis [38]. Monoterpenes have insecticidal properties, but the methods by which they work as natural insecticides are poorly understood. The possible targets related to their neurotoxic effects are positive allosteric modulators at insect Gamma-aminobutyric acid receptors [48,49,50]. Monoterpenes can inhibit acetylcholinesterase activity [51,52], but the inhibition of this enzyme is not likely the primary mode of action for carvacrol [53]. These effects cause paralysis and death in the insect. In addition, carvacrol and thymol can bind to the nicotinic acetylcholine receptor [50], and they are inhibitors of the transient receptor potential, essential components of biological sensors that detect changes in the environment in response to a myriad of stimuli [54].

Carvacrol and thymol also affect the reproductive system and show potential as ovicidal agents against *Cx. pipiens*, causing egg mortality by inhibiting egg hatching at LC_50_ values of 7 and 13 mg/L for carvacrol and thymol, respectively [43]. The mechanism by which phytochemicals cause egg mortality has been discussed. Dias et al., (2019) [46] reported that the ovicidal effects of carvacrol and thymol in the case of *M. spectabilis* are due to the chemicals passing through the egg pores that normally facilitate the passage of oxygen and small-molecule chemicals into the egg membrane. Consequently, the chemicals can be toxic to the insect egg [46]. In the present study, *P. amboinicus* essential oil caused ovarian necrosis, although this phenomenon was absent in *S. calcitrans*. Therefore, *P. amboinicus* essential oil might be effective against the reproductive system of some target insects. However, further research is needed to determine the exact mechanism, i.e., whether the insecticidal effect targets the ovary or the egg.

SEM results showed that *P. amboinicus* essential oil exerted no effect on the external morphology of *S. calcitrans* or *T. megalops*. These results were consistent with the effects of using goat weed (*Ageratum conyzoides*) essential oil on the external morphology of adult *Ae. aegypti* [18]. The absence of external morphological changes may be due to the fact that the lipophilic components in the essential oil can pass through the cuticle of the insect, causing more irritation and damage to internal tissues [55]. By contrast, a study on the effect of diatomaceous earth on *C. maculatus* and bean weevil (*Acanthoscelides obtectus*) revealed damage to the cuticle and ensuing rapid water loss, which is fatal to the insect [56].

## 5. Conclusions

Plant natural products, which are biodegradable and less toxic to humans and other mammals, are potent alternatives to synthetic insecticides in vector control programs. The results of contact and fumigant tests demonstrate that *P. amboinicus* essential oil has insecticidal properties against *S. calcitrans* and *T. megalops.* Histopathological findings showed the effects of the essential oil on the nervous and reproductive systems of the target insects. In future studies, flies taken from laboratory colonies should be tested to ensure that results are based on individuals with similar age and physiological status. Our results suggest that *P. amboinicus* could be developed into a natural insecticide to control stable flies and horse flies.

## Figures and Tables

**Table 1:** Percent mortality (mean ± SD) of insecticidal activity of *Plectranthus amboinicus* essential oil against *Stomoxys calcitrans* by contact toxicity test at different concentrations.

Concentration (µg/µL)	Mortality (%)					
	1 h	2 h	4 h	6 h	12 h	24 h
Negative control	0 ^a^	0 ^a^	0 ^a^	0 ^a^	0 ^a^	0 ^a^
Cypermethrin (1%)	100 ^d^	100 ^c^	100 ^c^	100 ^c^	100 ^d^	100 ^c^
9.3	0 ^a^	0 ^a^	13.3 ± 15.3 ^ab^	13.3 ± 15.3 ^a^	16.7 ± 11.6 ^ab^	26.7 ± 11.6 ^b^
18.7	3.3 ± 5.8 ^a^	3.3 ± 5.8 ^a^	6.7 ± 5.8 ^ab^	13.3 ± 15.3 ^a^	20.0 ± 10.0 ^ab^	33.3 ± 5.8 ^b^
37.4	16.7 ± 11.6 ^ab^	16.7 ± 11.6 ^ab^	23.3 ± 15.3 ^ab^	23.3 ± 15.3 ^ab^	30.0 ± 10.0 ^b^	50.0 ± 17.3 ^b^
46.7	33.3 ± 15.3 ^b^	36.7 ± 11.6 ^b^	36.7 ± 11.6 ^b^	50.0 ± 10.0 ^b^	63.3 ± 15.3 ^c^	80.0 ± 10.0 ^c^
93.4	70.0 ± 17.3 ^c^	73.3 ± 20.8 ^c^	73.3 ± 20.8 ^c^	83.3 ± 5.8 ^c^	83.3 ± 5.8 ^cd^	96.7 ± 5.8 ^c^
df	6, 14	6, 14	6, 14	6, 14	6, 14	6, 14
F	45.873	45.121	27.302	37.280	49.630	50.796
*p*-value	<0.001	<0.001	<0.001	<0.001	<0.001	<0.001

Statistically significant differences (*p* < 0.05) are indicated by different letters.

**Table 2:** Percent mortality (mean ± SD) of insecticidal activity of *Plectranthus amboinicus* essential oil against *Tabanus megalops* by contact toxicity test at different concentrations.

Concentration (µg/µL)	Mortality (%)					
	1 h	2 h	4 h	6 h	12 h	24 h
Negative control	0 ^a^	0 ^a^	0 ^a^	0 ^a^	0 ^a^	0 ^a^
Cypermethrin (1%)	100 ^b^	100 ^d^	100 ^d^	100 ^d^	100 ^d^	100 ^e^
93.4	0 ^a^	0 ^a^	0 ^a^	3.3 ± 5.78 ^a^	6.7 ± 5.8 ^a^	10.0 ± 0.0 ^a^
116.8	6.8 ± 5.8 ^a^	10.0 ± 0.0 ^ab^	13.3 ± 5.8 ^ab^	20.0 ± 10.0 ^ab^	43.3 ± 5.8 ^b^	43.3 ± 5.8 ^b^
140.1	10.0 ± 0.0 ^a^	20.0 ± 17.3 ^ab^	26.7 ± 20.8 ^abc^	26.7 ± 20.8 ^abc^	56.7 ± 5.8 ^bc^	60.0 ± 0.0 ^c^
163.5	16.7 ± 15.3 ^a^	33.3 ± 15.3 ^bc^	36.7 ± 11.6 ^bc^	43.3 ± 15.1 ^bc^	66.7 ± 11.6 ^c^	73.3 ± 5.8 ^d^
186.8	20.0 ± 10.0 ^a^	53.3 ± 15.3 ^c^	56.7 ± 15.3 ^c^	56.7 ± 15.3 ^c^	73.3 ± 5.8 ^c^	83.3 ± 5.8 ^d^
df	6, 14	6, 14	6, 14	6, 14	6, 14	6, 14
F	71.242	35.377	32.200	24.667	102.208	292.000
*p*-value	<0.001	<0.001	<0.001	<0.001	<0.001	<0.001

Statistically significant differences (*p* < 0.05) are indicated by different letters.

**Table 3:** Lethal dose (LD_50_ and LD_90_) and lethal concentration (LC_50_ and LC_90_) of *Plectranthus amboinicus* essential oil against *Stomoxys calcitrans* and *Tabanus megalops* by contact and fumigant toxicity tests at 24 h after treatment.

Treatment	Contact Toxicity Test		Fumigant Toxicity Test	
	*S. calcitrans*	*T. megalops*	*S. calcitrans*	*T. megalops*
LD_50_ [µg/fly] (95% CL)	12.05 (9.15–15.18)	131.41 (121.05–141.50)	-	-
LD_90_ [µg/fly] (95% CL)	45.53 (32.22–83.64)	200.62 (178.95–264.20)	-	-
LC_50_ [mg/L air] (95% CL)	-	-	1.34 (1.05–1.68)	7.12 (5.33–9.20)
LC_90_ [mg/L air] (95% CL)	-	-	4.39 (3.18–7.57)	30.37 (24.82–57.61)
Slope ± SE	2.21 ± 0.36	6.97 ± 1.14	2.49 ± 0.39	2.03 ± 0.91
χ^2^	7.74	1.52	2.94	3.85

95% CL = 95% confidence limit; S.E. = standard error; χ^2^ = chi-square.

**Table 4:** Percent mortality (mean ± SD) of insecticidal activity of *Plectranthus amboinicus* essential oil against *Stomoxys calcitrans* by fumigant toxicity test at different concentrations.

Concentration (mg/L Air)	Mortality (%)					
	1 h	2 h	4 h	6 h	12 h	24 h
Negative control	0 ^a^	0 ^a^	0 ^a^	0 ^a^	0 ^a^	0 ^a^
Cypermethrin (1%)	100 ^b^	100 ^b^	100 ^d^	100 ^b^	100 ^e^	100 ^c^
0.47	3.3 ± 5.8 ^a^	3.3 ± 5.8 ^a^	3.3 ± 5.8 ^ab^	6.7 ± 5.8 ^a^	6.7 ± 5.8 ^ab^	10.0 ± 10.0 ^a^
0.93	0 ^a^	0 ^a^	3.3 ± 5.8 ^ab^	26.7 ± 15.3 ^a^	26.7 ± 15.3 ^bc^	36.7 ± 23.1 ^ab^
1.87	0 ^a^	3.3 ± 5.8 ^a^	6.7 ± 5.8 ^ab^	26.7 ± 15.3 ^a^	36.7 ± 5.8 ^c^	63.3 ± 15.3 ^b^
2.80	3.3 ± 5.8 ^a^	3.3 ± 5.8 ^a^	16.7 ± 5.8 ^bc^	33.3 ± 25.2 ^a^	73.3 ± 5.8 ^d^	86.7 ± 5.8 ^c^
4.67	6.7 ± 5.8 ^a^	10.0 ± 0.0 ^a^	23.3 ± 5.8 ^c^	73.3 ± 15.3 ^c^	76.7 ± 11.6 ^d^	93.3 ± 11.6 ^c^
df	6, 14	6, 14	6, 14	6, 14	6, 14	6, 14
F	288.111	282.667	158.133	19.967	65.452	24.295
*p*-value	<0.001	<0.001	<0.001	<0.001	<0.001	<0.001

Statistically significant differences (*p* < 0.05) are indicated by different letters.

**Table 5:** Percent mortality (mean ± SD) of insecticidal activity of *Plectranthus amboinicus* essential oil against *Tabanus megalops* by fumigant toxicity test at different concentrations.

Concentration (mg/L Air)	Mortality (%)					
	1 h	2 h	4 h	6 h	12 h	24 h
Negative control	0 ^a^	0 ^a^	0 ^a^	0 ^a^	0 ^a^	0 ^a^
Cypermethrin (1%)	100 ^b^	100 ^b^	100 ^b^	100 ^c^	100 ^d^	100 ^d^
2.34	0 ^a^	0 ^a^	0 ^a^	3.3 ± 5.8 ^a^	10.0 ± 10.0 ^ab^	13.3 ± 11.6 ^a^
4.67	0 ^a^	0 ^a^	0 ^a^	0 ^a^	23.3 ± 11.6 ^b^	46.7±11.6 ^b^
9.34	0 ^a^	0 ^a^	0 ^a^	16.7 ± 5.8 ^b^	50.0 ± 0.0 ^c^	50.0 ± 0.0 ^b^
18.68	0 ^a^	0 ^a^	0 ^a^	3.3 ± 5.8 ^a^	46.7 ± 11.6 ^c^	76.7 ± 5.8 ^c^
28.02	3.3 ± 5.8 ^a^	6.7 ± 11.6 ^a^	6.7 ± 11.6 ^a^	16.7 ± 5.8 ^b^	86.7 ± 15.1 ^d^	93.3 ± 5.8 ^cd^
df	6, 14	6, 14	6, 14	6, 14	6, 14	6, 14
F	891.000	221.000	221.000	204.167	79.611	92.567
*p*-value	<0.001	<0.001	<0.001	<0.001	<0.001	<0.001

Statistically significant differences (*p* < 0.05) are indicated by different letters.

## Data Availability

The data presented in this study are available within the article.
